# Translating a Home-Based Breathlessness Service: A Pilot Study of Feasibility, Person-Reported, and Hospital Use Outcomes

**DOI:** 10.3390/jcm14113894

**Published:** 2025-06-01

**Authors:** Kylie N. Johnston, Mary Young, Debra Kay, Marie T. Williams

**Affiliations:** 1 Implementation and Clinical Translation in Health (IIMPACT), Allied Health and Human Performance, University of South Australia, Adelaide 5000, Australia; marie.williams@unisa.edu.au; 2 Department of Thoracic Medicine, Heart and Lung Service, Central Adelaide Local Health Network, Adelaide 5000, Australia; mary.young@sa.gov.au; 3 Independent Researcher, Adelaide 5000, Australia

**Keywords:** breathlessness, COPD, carers, accessibility, translation

## Abstract

**Background/Objectives:** Persistent breathlessness impacts people living with advanced chronic obstructive pulmonary disease (COPD) and carers. Accessible services are limited. This translational pilot study evaluated the feasibility, impacts on patient and carer-reported outcomes, and hospital use of a home-based breathlessness intervention service (BLIS). **Methods**: People with stable COPD, ≥1 COPD-related hospital admissions in the previous year, and persistent breathlessness participated in a pre–post study. The BLIS program involved home visits/phone contacts by a nurse/physiotherapist (average 8 contacts, 7 weeks). Uptake, retention, and fidelity were recorded prospectively, and participant experience was explored (post-program interviews). Breathing discomfort (Multidimensional Dyspnea Profile A1 scale), threat (Brief Illness Perception Questionnaire), and carer stress/strain (Zarit Burden Interview) were compared pre- and post-program (week 9, 3 and 6 months) using mean difference and 95% confidence intervals (CIs). Hospital use for COPD-related causes in 12 months before/after participation was reported. **Results**: A total of 16/19 eligible people agreed to participate, and 15/16 completed the program. In participants with COPD (73 [9] years, FEV1%pred 42% [15], mean [SD]; a median of 3 COPD-related hospital admissions in the previous year) and carers (*n* = 6), BLIS was highly (in 95%) acceptable. Compared to pre-program, breathing discomfort was reduced in week 9 and 6 months; breathlessness threat was reduced in week 9 and 3 months; and carer burden was reduced at 6 months. Compared to the 12 months prior, hospital admissions decreased in the 12 months post-program. **Conclusions**: Translation of this service to the local setting was feasible, with high program uptake and retention. Post-program improvements in key patient- and carer-reported outcomes and a reduction in public hospital admissions support the implementation of the BLIS program for this cohort in this setting.

## 1. Introduction

Breathlessness commonly persists in people with advanced and severe respiratory illnesses, including chronic obstructive pulmonary disease (COPD), even when guideline-based treatment is in place [1,2,3]. Impacts include reduced physical function, leading to disability, and psychosocial distress, including anxiety, depression, a loss of dignity, and a lack of understanding of the problem by others [4]. Carers report experiencing helplessness, uncertainty, and unmet support needs [5]. Hospital-related costs in people with advanced chronic disease and breathlessness are high, with average estimates for hospital care of GBP 1084/month per person, increasing to GBP 3836/month per person when unpaid care provided by family and friends was included [6].

Breathlessness services are a therapeutic model developed to reduce this health and financial impact [1,7]. Services typically use a holistic approach encompassing needs-based, mostly non-pharmacological interventions; incorporate skills from multiple health professional domains; and support self-management [7]. Systematic reviews [7,8] support the beneficial outcomes of breathlessness intervention services, although a lack of blinding and high loss to follow-up limit evidence certainty [8], and clinical guidelines suggest their use in people with serious respiratory illness [9]. Cost-effectiveness studies of breathlessness services have demonstrated a better quality of life (adjusted mean differences of QALY gains) at a non-significantly higher cost compared with usual care [10].

Home-based breathlessness intervention services were developed and commenced in the UK [11] at least 20 years ago and have been adapted into mainly clinic-based delivery formats, including in Germany [12], the Netherlands [13], Ireland [14], and reported on in Australia (Melbourne, clinic based [15], and Sydney, combination home/clinic [16]). There are currently no specific breathlessness intervention services in South Australia.

This study aimed to evaluate the feasibility and outcomes of translating an evidence-based breathlessness intervention service (BLIS) for people in the local setting with persistent breathlessness associated with COPD. To achieve this overall aim, this study had three specific objectives, which were the following:

1. Examine the feasibility and participant experience of the BLIS program;
2. Describe pre–post BLIS program changes in patient-reported outcomes of distress due to breathlessness and carer-reported outcomes of burden and wellbeing;
3. Describe public hospital-related health care utilization for a COPD-related cause 12 months before and after commencing the BLIS program.

## 2. Materials and Methods

### 2.1. Study Design and Ethics Approvals

This study employed a single group pre–post feasibility design hosted within a hospital in metropolitan Adelaide, South Australia. Data were collected during 2019. Ethics approval was granted by the Human Research Ethics Committee (HREC) of the Southern Adelaide Local Health Network (5 July 2018, 18/SAC/62) and the University of South Australia HREC (26 July 2018, 201493). This study was prospectively registered with the Australian New Zealand Clinical Trials Registry (9 January 2019, ACTRN12619000020189). Design and reporting were informed by guidelines for reporting non-randomized pilot and feasibility studies [17].

### 2.2. Consumer and Stakeholder Involvement

In response to an expression of interest for people with lived experience of breathlessness distributed in partnership with local support groups and health services, two consumer representatives were recruited to participate in the project design and delivery over 12 months. A wider project advisory group comprised nine representatives of relevant local, national, and international organizations.

### 2.3. Participant Eligibility Criteria

People living with chronic breathlessness were eligible if they (1) had a diagnosis of COPD, were receiving recommended management [18], and had a minimum of one respiratory-related hospital admission in the past year; (2) were troubled by breathlessness; (3) lived within the local health network area; and (4) were at least four weeks after discharge from hospital/in a stable state at the time of commencing the pilot study. Carers were eligible if they provided unpaid support on a regular basis to a person participating in this study. People living with COPD accessing case management from other programs or receiving end-of-life palliative care services were ineligible.

### 2.4. Study Recruitment

Study information and eligibility requirements were provided to medical and nursing staff. A clinician member of the study team (Respiratory Nurse Practitioner (RNP), MY) reviewed hospital administrative data to identify individuals who potentially met eligibility criteria. The RNP contacted potential participants by phone and, where indicated, mailed study information. In a follow-up phone call one week later, if potential participants indicated a willingness to be involved, eligibility criteria were confirmed, and a pre-program home visit date was negotiated. At this pre-program home visit, the RNP completed an organization-standard offsite visit risk assessment, explained this study in more detail, and clarified any areas of uncertainty. Where potential participants accepted the invitation to join, written consent was obtained, and pre-program questionnaire booklet/s were provided. Where potential participants declined, usual medical care was continued.

### 2.5. Breathlessness Intervention Service (BLIS) Program Intervention

The BLIS program intervention was prospectively planned to be voluntary, free of cost to consumers, conducted over eight weeks comprising 2–3 home visits (RNP and/or physiotherapist) with follow-up support by 2–3 phone calls (Table S1). The BLIS program was based on the Cambridge Breathlessness Intervention Service (CBIS) [19], with the Breathing Thinking Functioning (BTF) clinical model [20] used to assess and prescribe evidence-based non-drug strategies based on an individual’s presenting symptoms. Educational resources were reproduced with kind permission from the CBIS. An individualized breathlessness action plan (adapted from [15], Supplement S1) was created in collaboration with and provided to each participant.

The BLIS intervention was provided in conjunction with support by care coordination, facilitation of self-management (identifying breathlessness-related problems and progress toward goals [21]), and integration with other health and community service providers. Discussion with carers regarding needs and support was conducted incorporating the Carer Support Needs Assessment Tool [22].

### 2.6. Program Feasibility and Acceptability

The primary outcome of this pilot study concerned the feasibility and participant acceptability of the BLIS program. Feasibility criteria were established a priori (Table 1). Fidelity was assessed for congruency with key published elements of the CBIS approach [19] using a pre-developed 15-item template, using a video recording of a session. Acceptability was based on the National Health Service (NHS) Friends and Family Test question, “Having thought about your experience with the BLIS program, how likely are you to recommend our program to friends and family if they needed similar care or treatment?” [23], which formed part of the post-program phone-based interviewer-administered survey (criterion = “likely” or “extremely likely” to recommend in at least 80% of participants).

### 2.7. Person Living with Breathlessness/Carer-Reported Outcomes

Questionnaires provided to the person living with breathlessness and carers are summarized in Table 1 (detail Table S2). One week after the pre-program home visit, a study team member not involved in recruitment or program delivery (MTW) phoned each participant to confirm receipt of the questionnaire package, enquire whether there had been a self-completed, prompt return of questionnaires in the reply-paid envelope provided and/or support interviewer-assisted completion by reading each questionnaire item and response options, and marking responses in an interviewer copy. The same process was used on each occasion of data collection (post-BLIS program at one week and 3 and 6 months). The same team member completed a post-program phone-based interviewer-administered survey. People living with breathlessness and carers were interviewed separately. Survey questions included open-response questions (best aspects, areas for improvement, use of resources) and ratings for overall program acceptability [23].

### 2.8. Hospital-Related Health Care Utilization

The number of public hospital admissions, length of stay, and emergency department presentations without overnight admission [30] for a COPD-related cause in the 12-month periods before and after the commencement of the BLIS program were recorded from hospital clinical information systems. The number of hospital admissions for people with COPD included occasions when people presented to the hospital through the emergency department for a COPD-related cause, and this resulted in hospital admission. The number of emergency department presentations included occasions where people presented to the emergency department with a COPD-related cause, and this did not result in an overnight admission. Costs of public hospital presentations and admissions were estimated using hospital-assigned diagnosis-related group (DRG) 2018–2019 cost figures [31]. People who completed the BLIS program but died in the 12 months following the program were excluded from this analysis.

### 2.9. Data Management and Synthesis

Participant characteristics were reported descriptively (frequencies and percentages, mean and standard deviation [SD] for continuous data). Feasibility criteria (Objective 1) were reported descriptively (frequencies and percentages) according to stated definitions and a priori criterion thresholds (Table 1). Transcripts of post-program interviews were examined using qualitative content analysis to summarize participant feedback. To describe changes in self-reported outcomes after participation in the BLIS program (Objective 2), questionnaires were scored as per the developers’ recommendations and reported descriptively (mean [SD] for normally distributed data, median and interquartile range [IQR] for non-normally distributed data). Mean difference and a 95% confidence interval of the difference from pre-program scores in self-reported outcomes at one week and 3 and 6 months post-program, using all matched data available at the time points, were reported. Differences from the baseline where the 95% confidence interval did not cross zero were identified as an indicator of potential effectiveness, with the 95% confidence interval reported as a measure of precision [17]. Public hospital-related health care utilization for a COPD-related cause (emergency department presentations, hospital admissions, hospital length of stay, and DRG-based cost estimations) in the 12 months before and after commencement in the BLIS program were reported descriptively (frequencies and percentages) (Objective 3). All descriptive statistics were analyzed using IBM SPSS Statistics Version 28.0.0.1.

## 3. Results

### 3.1. Participant Baseline Characteristics

**Figure 1:**
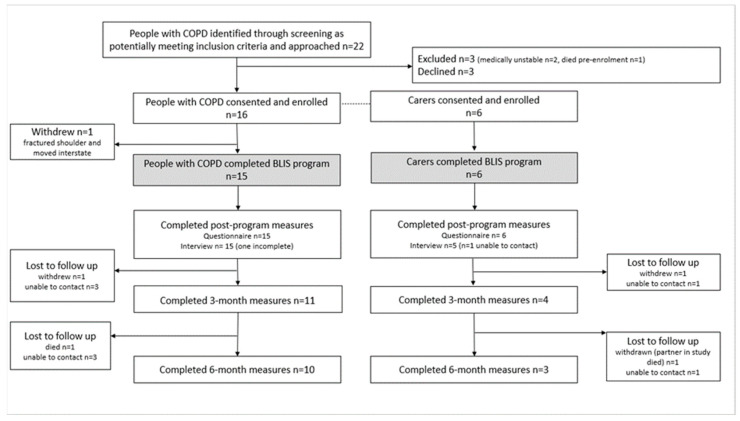
Participant recruitment and flow through this study.

Participant flow is presented in Figure 1. Fifteen people with chronic breathlessness and six carers participated (Table 2). All participants living with breathlessness had multiple (average of nine) documented comorbid medical conditions, and most were prescribed long-term oxygen therapy. All carers were spouses and lived with the respective participants. No other demographic information was gathered from carers.

### 3.2. BLIS Program Delivery

Characteristics of the BLIS program delivery (Table 3) and interventions provided are summarized (Table 4). Where issues related to COPD chronic disease management (Table 4) were identified during assessment, these were addressed prior to or concurrent with the delivery of other aspects of the BLIS program. Exacerbation management at home and/or facilitating urgent medical review resulted in avoidance of hospital use in 11 instances in 9/15 participants.

### 3.3. Feasibility and Acceptability Results: Study Objective 1

The BLIS program met all a priori criteria (Table 1) for feasibility and acceptability.

Program uptake and retention: Of the twenty-two potentially eligible people with COPD, three were ineligible; nineteen were invited and three declined, resulting in program uptake of 84% (16/19, Figure 1). When participants identified a carer (*n* = 6), all joined the program. Immediate post-program measures were completed by 94% (15/16) of original participants with COPD and 83% (5/6) of carers.

Outcome measure completion: Pre-program completion was 100% in four of six measures (MDP A1 scale, CRQ-SAS, B-IPQ, DASS21) and 93% in the other two (AQoL8-D, ability to manage, Table S3). Immediate post-program completion was 100% in five of six measures and 87% in the remaining measure (B-IPQ, Table S4). Carer questionnaires were fully completed at pre- and post-program time points.

Program fidelity: One 60 min intervention visit was examined for congruency. All key elements (*n* = 15) were observed either in the initial BLIS program assessment visit (14/15) or identified in participant responses to the pre-program questionnaires (1/15).

Participant experience and program acceptability: Post-program interviews were conducted with all people with COPD (*n* = 15) and five of the six carers (Figure 1). Ninety-five percent of individuals (19/20) reported that they were likely or extremely likely to recommend this program. Participants indicated that the BLIS program had a significant impact (70%, 14/20 participants), some impact (25%, 5/20 participants), or no impact (5%, *n* = 1). Common themes in responses to interview questions are shown in Table 5.

### 3.4. Person Living with Breathlessness/Carer-Reported Outcome Results: Study Objective 2

Key outcomes in people living with breathlessness: Compared with the pre-program baseline, breathing discomfort (MDP A1 scale) was lower post-program (mean difference −1.0 points, 95% CI for the difference −2.0 to −0.1, Table 6) and at six months (−1.1, 95% CI −2.1 to −0.1). The MDP Emotional Response item for anxiety decreased post-program (mean difference −1.5 points,95% CI −1.5 to −3.7 Table S4). Consistent with these results were reductions (Table S5) in the perception of breathlessness threat (BIPQ-B total score post-program −5.1 points, 95% CI of difference −9.7 to −0.5 points; 3-month −7.2, 95% CI −12.9 to −1.5) and anxiety (anxiety subscale DASS-21 post-program −4.1 points, 95% CI −7.5 to −0.8) and improvements in the AQOL-8D coping dimension score (post-program −1.1, 95% CI −2.0 to −0.3; 3-month −1.2, 95% CI −2.3 to −0.7).

Perceived control of breathlessness (CRQ mastery) and ability to manage/live with breathlessness (VAS score 0–10) were relatively high at the baseline, skewed positively, and unchanged during follow-up (Table 6). Scores in total AQOL-8D and other DASS-21 domains (Stress, Depression) were unchanged (Table S5).

Key outcomes in carers: Six carers completed immediate post-program measures (3-month measures *n* = 4; 6 month *n* = 3, Figure 1). Pre-program, the mean ZBI-12 score was 17.6 points. Scores did not change post-program (Table 7); although, for carers who remained in this study at 6 months, mean scores were reduced by 7 (95% CI −13.6 to −0.4). Pre-program scores on all subscales of the DASS-21 were low (non-clinical ranges [32]) and remained mostly unchanged during the 6-month post-BLIS period. The exception was the stress subscale, which increased at 3-month follow-up (by 4.0 points, 95% CI of difference 1.4 to 6.6), although this was not consistent with change scores associated with clinical worsening [32]. Carers identified needs for understanding illness and knowing what to expect in the future; dealing with emotions; accessing services for themselves and the person they supported; and communicating with health professionals (needs, actions taken, Supplement S2).

### 3.5. Hospital-Related Health Care Utilization Results: Study Objective 3

One participant died in the 12 months following the program and was excluded from this analysis (Table 8). The total number of hospital admissions among the participants reduced from 47 in the year before the BLIS program to 18 in the year after (62% reduction). The total COPD-related hospital length of stay for this cohort was 270 days in the year before the program and 89 days in the year after (67% reduction). Estimated public hospital use costs for COPD exacerbations (hospital admissions plus emergency department presentations) in the 12 months before and after the BLIS program were AUD 502,200 and AUD 169,856, respectively, a net reduction of AUD 332,344.

## 4. Discussion

The key findings of this translational pilot study were that the BLIS program (1) was feasible, acceptable, and accessible to a group of people with COPD with previous high hospital use; (2) implemented more frequent visits than anticipated to optimize COPD disease management; (3) comprehensively delivered non-pharmacological breathlessness management strategies and supported carers; (4) had a positive impact on lowering distress and perceived threat associated with breathlessness; and (5) participants’ hospital use related to COPD was lower in the 12 months after than 12 months before the program.

Having this service provided at home was identified (by 13/15 participants) as the most helpful aspect of program delivery, contextualizing every aspect of the person’s everyday life. High uptake and retention indicate that the program was able to be accessed by this cohort with breathlessness and complex needs. The findings confirm the value of tailored home-based interventions, recognized in early qualitative studies from the development of the CBIS [33] to recent client perspectives on how to optimize a breathlessness service [16].

While two or three home visits were planned, an average of 6.6 visits were conducted, with early visits in some cases related to optimizing COPD chronic disease management. Diagnosis and optimized treatment of the underlying medical conditions may be considered a starting point for breathlessness service management, to which additional strategies are added [1,3]. Although participants of the BLIS program had at least three hospital admissions for COPD in the previous year, disease education, inhaler device assessment, and exacerbation management education were frequently indicated.

Over the course of the BLIS program, two (in 13/15 participants, 87%) or three of the BTF cycles (in 2/15, 13%) were identified as contributing. “Breathing” was most often identified (87% of cases), followed by “Functioning” (73%) and “Thinking” (53%). Strategies implemented most frequently (80–100% of participants) were the hand-held fan, body positioning, breathing strategies, and individualized home exercise programs. Specific strategies associated with the “Thinking” cycle were less often reported (less than or equal to 60% of cases). This may reflect the use of integrated and applied implementation of psychological strategies that reduce fear associated with breathlessness and increase self-efficacy to manage breathlessness during activity [34]. These may be “unlabeled” [35] parts of the therapeutic application by trained health professionals that occur, for example, when supporting a breathless person to use the fan or breathing techniques while exercising.

Delivering the BLIS program at home provided an opportunity for carers to receive comprehensive assessment and intervention side by side with the person with breathlessness. Carer stress and strain at entry to the BLIS program were high, with ZBI-12 scores exceeding those reported by carers supporting people with advanced cancer and dementia [36]. Expressed needs were complex and varied from education and emotional support to direct service needs, consistent with previous studies [5,37]. Carers have identified that receiving professional support at the time of their patient’s breathlessness would have been helpful [5] and that qualities of rapport, continuity, not being rushed, and delivery in a non-hospital setting were important [37]. These aspects were all present in the BLIS program, supporting its suitability as a service delivery platform for carers.

Post-BLIS program and at six months, mean changes from baseline in severity of breathing discomfort (MDP A1 score) were clinically meaningful, with improvements that exceeded the minimally important difference for this score over similar time periods [38]. While not directly comparable due to differences in study design and instruments, this direction of change concurs with immediate (4–8 weeks) reductions in numerical rating scales of breathlessness severity in a meta-analysis of randomized controlled trials of multicomponent breathlessness services [7].

In the 12 months after participation in the BLIS program, participants in this study cohort had fewer interactions with the hospital via the emergency department overall (accounting for interactions with the emergency department that did and did not result in hospital admission, i.e., 33 versus 49 occasions, Table 8) than in the previous 12-month period. This suggests that, overall, participants may have been more confident and better able to manage symptoms at home. After participation in the BLIS program, far fewer of those interactions with the hospital service were severe enough to require a hospital admission (18 versus 47 admissions), and thus post-BLIS program, three more emergency department presentations only, without overnight admission, were recorded, than seen pre-BLIS.

The 12-month post-program follow-up period for our participants included an average of 5.3 months (SD 2.7, range 1–8 months) after the onset of the COVID-19 pandemic (March 2020). This time was associated globally with reduced admissions for COPD exacerbations (50% pooled rate ratio reduction in a meta-analysis of nine studies [39]), although admission rates in people with multiple comorbidities and recurrent admissions, such as our participants, were less affected by COVID-19 [40]. The impact of COVID-19 public health measures may partly explain some of the reduction in hospital admissions during this time.

Limitations: This was a translational pilot study with no control group. Consistent with the guidelines for the reporting of non-randomized pilot studies [17], the group mean difference and the 95% confidence interval of that difference in self-reported outcome measures from the baseline after participation in the program were reported descriptively. Where confidence intervals of difference did not include zero and moved in the direction of improvement, we can be 95% confident that the change was not equal to zero. These findings indicate potential effectiveness but should be interpreted with caution, taking into consideration the single-group study design. Recruitment involved a single center, generating a small sample from a specific area, so the results may not be generalizable to other contexts and health care systems. While participant numbers, especially carers, declined at the six-month measures, this is one of few breathlessness service studies to describe any carer self-reported outcomes [12] or follow-ups longer than the immediate post-program period [7].

## 5. Conclusions

Translation of the home-based CBIS model was feasible, acceptable, and accessible to people living with advanced COPD (patients and carers). Post-program changes in participant breathing discomfort, breathlessness threat, and carer burden indicate potential effectiveness, continuing up to six months after program completion. The reduction observed in hospital admissions 12 months post-program contrasts with the expected upward trajectory for this cohort. The findings support the ongoing implementation of the BLIS program for this cohort in this setting, combined with the evaluation of selected outcome measures and cost–benefit analysis.

## Figures and Tables

**Table 1:** Summary of pilot data collected arranged by study objectives and occasion of assessment.

Objective 1: Feasibility and Acceptability		Week 0	BLIS	Week 9	3 mths	6 mths
Program uptake	Number consented as a % of those eligible and approached [75% uptake] ^a^	X				
Program retention	Number completing post-program assessment as a % of those who commenced [80% retention] ^a^			X		
Measure completion	Number of completed questionnaires (pre- and post-program) as a % of questionnaires [75% completed] ^a^			X		
Program fidelity	Videoed assessment/intervention versus CBIS approach [19] [75% congruent] ^a^		X			
Program acceptability	NHS Family and Friends Test [23] [80% positive experience] ^a^; semi-structured telephone interview			X		
**Objective 2: Person living with breathlessness/carer reported outcomes**						
Domain	Instrument					
Breathlessness discomfort	Multidimensional Dyspnoea Profile (A1 scale) [24]	X		X	X	X
Perceived control of breathlessness	CRQ Self-Administered (mastery subscale) [25]	X		X	X	X
Manage/live with breathlessness	Visual Analog Scale (0 = not able to manage at all, 10 = able to manage extremely well)	X		X	X	X
Breathlessness threat	Brief Illness Perception Questionnaire (breathlessness version) [26]	X		X	X	X
Depression, anxiety, and stress	Depression, Anxiety, and Stress Scale (DASS-21) [27]	X		X	X	X
Quality of Life	Assessment of Quality of Life (AQoL-8D) [28]	X		X	X	X
Carer burden	Zarit Burden Interview (ZBI-12) [29]	X		X	X	X
Managing/living with their relative’s breathlessness	Visual Analog Scale (0 = not able to manage at all, 10 = able to manage extremely well)	X		X	X	X
Depression, anxiety, and stress	Depression, Anxiety, and Stress Scale (DASS-21) [27]	X		X	X	X
**Objective 3: Hospital-related health care utilization**						
Health care use	12 months before and after the program Number of hospital admissions and length of stay ^b^ Number of emergency department presentations ^b^ Cost estimates based on the diagnosis-related group ^b^	←				→

X indicates data collected at this occasion of assessment; ← indicates data collected in the 12 months before the program;→ indicates data collected in the 12 months after the program. ^a^ Priori criterion threshold for feasibility; ^b^ COPD-related. BLIS = breathlessness intervention service; CBIS = Cambridge Breathlessness Intervention Service; CRQ = Chronic Respiratory Questionnaire; mths = months; NHS = National Health Service.

**Table 2:** Characteristics of participants: people living with breathlessness, *n* = 15.

	Frequency (%) or Mean (SD)
Demographic features	
Gender, male/female	7 (47)/8 (53)
Age, years	73 (9)
Living arrangements	
Alone	7 (47%)
With spouse	6 (40%)
With other family/friend	2 (13%)
Housing arrangements	
Lived in their own home	9 (60%)
Rented accommodation	6 (40%)
Primary respiratory diagnosis	
COPD	10 (67%)
Asthma/COPD overlap	5 (33%)
Lung function test results, post-bronchodilator (*n* = 14)	
FEV_1_% predicted	42 (15)
FVC% predicted	64 (25)
FEV_1_/FVC%	44 (9)
Prescribed long-term oxygen therapy	
Yes	10 (67)
No	5 (33)
Modified Medical Research Council Dyspnea Scale (*n* = 14)	
0: “I only get breathless with strenuous exercise”	0
1: “I get short of breath when hurrying on the level or walking up a slight hill”	0
2: “I walk slower than people my own age on the level because of my breathlessness or have to stop for breath when walking at my own pace on the level”	5 (33)
3: “I stop for breath after walking about 100 yards or after a few minutes on the level”	10 (67)
4: “I am too breathless to leave the house”	0
Comorbidities	
Number of comorbid conditions per participant	9 (3)
Mental health comorbidities	
Depression	7 (47)
Anxiety	4 (27)
Number of current medications at study commencement	9(4)
Polypharmacy (5 or more medications)	14 (93)
Hospital encounters in the previous 12 months	median (IQR)
COPD-related emergency department presentations	0 (0, 1)
COPD-related admissions	3 (2.5, 3)
COPD-related total length of stay (days)	15 (10.5, 31.5)

COPD = chronic obstructive pulmonary disease; FEV_1_ = forced expiratory volume in one second; FVC = forced vital capacity, IQR = interquartile range, SD = standard deviation.

**Table 3:** BLIS program delivery characteristics; *n* = 15 participants with COPD.

		Mean (SD)/*n* (%)
BLIS program visits	Number of therapist home visits per participant	6.6 (1.5)
	Duration of therapist home visit (minutes)	63 (8)
	Total therapist contacts per participant	7.6 (2.1)
	Program duration (weeks)	6.7 (2.2)
Program-related non-clinical time ^a^ by respiratory NP, hours per participant		5.2(1.7)
Types of contacts provided in the BLIS program, % of the total of contacts *n* = 109		
	Respiratory NP individual home visit	67 (61%)
	Physiotherapist individual home visit	19 (17%)
	Joint respiratory NP/physiotherapist home visit	8 (7%)
	Telephone call	10 (9%)
	Outpatient attendance	5 (5%)

^a^ Program-related correspondence, clinical history and investigation gathering, participant-specific documentation; BLIS = breathlessness intervention service; NP = nurse practitioner, SD=standard deviation.

**Table 4:** BLIS program intervention strategies delivered; *n* = 15 participants with COPD.

Intervention Strategy		Mean (SD)/*n* (%)
Breathing/Thinking/Functioning Intervention strategies		
Breathing strategies	Hand-held fan	15 (100%)
	Fan/forward lean or flop/focus on the breath out	15 (100%)
	Body positioning	13 (87%)
	Breathing exercises	13 (87%)
	Airway clearance	5 (33%)
Thinking strategies	Challenging unhelpful thoughts	9 (60%)
	Managing expectations	5 (33%)
	Managing anxiety	4 (27%)
	Self-talk/mantra for recovery	4 (27%)
	Mindfulness/relaxation script	2 (13%)
Functioning strategies	Home exercise program	12 (80%)
	Energy conservation	9 (60%)
	Pedometer program/use	8 (53%)
	Priority diary	1 (7%)
BLIS program resources use		
	Individualized breathlessness action plan	15 (100%)
	BLIS program toolkit booklet	13 (87%)
	Videos (St Christophers’ ^a^/Cambridge BIS ^b^)	13 (87%)
Disease management strategies		
	Disease education	12 (80%)
	Disease-printed resources	10 (67%)
	Inhaler device assessment	9 (60%)
	Coordinate health and social care services	4 (27%)
	Advance care planning discussion/printed resource	4 (27%)
	Symptom diary	1 (7%)
	Nutrition education	1 (7%)
COPD exacerbation management strategy		
	Exacerbation management education	10 (67%)
	Home exacerbation management	7 (47%)
	Facilitate urgent GP/respiratory physician review	7 (47%)

^a^ Publicly available resource at https://www.stchristophers.org.uk/breathlessness-management/ (accessed 1 May 2025); ^b^ publicly available resource at https://www.cuh.nhs.uk/our-services/breathlessness-intervention-service/video-and-audio-help-manage-breathlessness/ (accessed 1 May 2025). BLIS = breathlessness intervention service; GP = general practitioner.

**Table 5:** Post-program interview (*n* = 20) responses about the impacts and value of the BLIS program.

Common Themes from Interview Responses	Number of Responses
Impacts of the program	
Changed the way I cope with breathlessness; I’m more in control	11
More confident and not as frightened, worried, or panicked	11
Able to do more of what matters to me	6
Made a positive difference in relationships between person and carer	4
How BLIS made that impact/most helpful aspects of the BLIS program	
Using the fan	13
Changes to breathing and posture	13
Changed the way I think	10
Now I know something can be done; what to do to cope	3
Walking/exercising more	13
Using the BLIS toolkit and resources	6
Using oxygen and medicines more effectively now	5
Helpful aspects of the way the BLIS program was delivered	
Program being at home ^a^	13
Positive qualities of the care provided ^b^	9
The way things were explained ^c^	6
Length of time and frequency of visits	6
Working with both the person and the carer	3
Links to and provision of other services	1
Doing questionnaires over the phone ^d^	1
Unhelpful aspects of the BLIS program	
Nothing	16
Doing questionnaires over the phone ^e^	1
Might be helpful for someone newly diagnosed	1
Areas for improvement	
Nothing	17
Think about getting people together ^f^	1

^a^ More relaxed, comfortable, more time, take in information better, avoid hospital visits, which are costly, exhausting, and difficult; ^b^ flexible, encouraging without being intrusive, validating, non-judgmental, knowledgeable, and caring; ^c^ giving information, talked through things, helped me relax, straightforward, and explained how and why; ^d^ having someone go through these with me; ^e^ would have been better face to face; ^f^ we do not get to see everyone on the BLIS program.

**Table 6:** Breathlessness discomfort, perceived control, and ability to manage scores in people living with breathlessness at pre-program compared with post-program and follow-up (3 and 6 months).

	Pre-Program *n* = 15	Post-Program *n* = 15	Difference Post–Pre 95% CI *n* = 15	3 mth Post-Program *n* = 11	Difference 3 mth–Pre 95% CI *n* = 11	6 mths Post-Program *n* = 10	Difference 6 mth–Pre 95% CI *n* = 10
**MDP A1 scale ^a^**	4.9 (2.3)	3.9 (1.8)	**−1.0 (1.8)** **−2.0, −0.1**	4.7 (2.4)	−1.2 (1.9) (−2.5, 0.1)	5.0 (2.1)	**−1.1 (1.4)** **−2.1, −0.1**
**CRQ mastery ^b^**	5.2 (1.6)	5.4 (1.2)	0.3 (1.4) −0.5, 1.1	5.2 (1.2)	−0.1 (0.9) −0.7, 0.5	5.2 (1.2)	0.3 (1.7) −1.0, 1.4
**Perceived ability ^c^** to manage/live with breathlessness in the last week	8 (4) *n* = 14, med (IQR)	8 (3) med (IQR)	0.1 (2.1) −1.1, 1.2 *n* = 14	8 (2) med (IQR)	0.7 (1.7) −0.4, 1.8	8 (3) med (IQR)	0.9 (1.9) −0.6, 2.4 *n* = 9

Bold text indicates that the 95% confidence interval of difference did not cross zero. Scores are reported as mean (SD) unless otherwise indicated. ^a^ 0 = neutral, 10 = unbearable; focal period = average over the last 2 weeks; ^b^ subscale range 1–7, higher is better; ^c^ Visual Analog Scale, 0–10, higher is better.CI = confidence interval; CRQ = Chronic Respiratory Questionnaire; IRQ = interquartile range; MDP = Multidimensional Dyspnea Profile; med = median; mth = month.

**Table 7:** Carer-reported outcomes: pre-program, post-program, and follow-up (3 and 6 months post-program).

	Pre-Program *n* = 6	Post-Program *n* = 6	Difference Post–Pre 95% CI *n* = 6	3 mths Post-Program *n* = 4	Difference 3 mth–Pre 95% CI *n* = 4	6 mths Post-Program *n* = 3	Difference 6 mth–Pre 95% CI *n* = 3
**Carer burden** Zarit Burden Interview	17.6 (7.4)	13.7 (10.9)	−4.0 (4.4) −8.6, 0.6	17.5 (9.0)	0 (2.7) −4.3, 4.3	8.6 (8.0)	**−7 (2.6)** **−13.6, −0.4**
**Perceptions of managing breathlessness**							
Rate the ability of the person you are caring for to manage/live with their breathlessness ^a^	6.7 (1.5)	6.0 (2.5)	−0.7 (3.0) −3.8, 2.5	5.0 (2.3)	−1.5 (3.1) −6.4, 3.4	6.7 (2.9)	0.3 (1.5) −3.5, 4.1
Rate your own ability to manage/live with the person you are caring for with breathlessness ^a^	7.5 (0.5)	7.0 (3.2)	−0.5 (3.0) −3.7, 2.7	6.3 (2.9)	−1.0 (2.4) −4.9, 2.9	8.3 (1.5)	0.7 (1.5) −3.1, 4.5
**DASS21**	Pre-Program med (IQR) *n* = 6	Post-Program med (IQR) *n* = 6	Difference Post–Pre 95% CI *n* = 6	3 mth Post-Program med (IQR) *n* = 4	**Difference** **3 mth–Pre** 95% CI *n* = 4	6 mths Post-Program med (Range) *n* = 3	**Difference** **6 mth–Pre** 95% CI *n* = 3
Depression subscale	2.0 (11.0)	2.0 (13.0)	1.0 (2.8) −1.9, 3.4	5.5 (13.0)	2.3 (2.1) −1.0, 5.5	0 (6)	−2.0 (2.0) −7.0, 3.0
Anxiety subscale	1 (7)	3 (9)	0.6 (5.8) −5.4, 6.7	5.0 (9.0)	3.5 (4.1) −3.1, 10.1	0 (10)	−2.7 (3.1) −10.3, 4.9
Stress subscale	9 (11)	3 (21)	0.7 (8.4) −8.1, 9.4	10.0 (11.0)	**4.0 (1.6)** **1.4, 6.6**	8 (12)	2.0 (5.3) −11.1, 15.1

Bold text indicates that the 95% confidence interval of difference did not cross zero. Scores are reported as mean (SD) unless otherwise indicated. ^a^ Visual Analog Scale, 0–10, higher is better; CI=confidence interval; DASS21 = Depression, Anxiety, and Stress Scale; IQR = interquartile range; med = median; mth = month; NRS = numerical rating scale.

**Table 8:** Public hospital-related use data for participants who completed the BLIS program, excluding one deceased participant (*n* = 14).

	Pre-BLIS Program (12 Months)	Post-BLIS Program (12 Months)
Hospital admissions for COPD		
Number of participants who were admitted (*n*, %)	14/14 (100%)	8/14 (57%)
Total number of admissions ^a^	47	18
Total length of stay (days)	270	89
Average length of stay per admission (days)	5.7	5.6
Total hospitalization cost (AUD)	AUD 500,676	AUD 163,756
Emergency department presentations for COPD		
Number of participants who presented (*n*, %)	2/14 (14%)	3/14 (21%)
Total number of presentations	2	5
Total presentation costs (AUD)	AUD 1524	AUD 6100

^a^ The total number of admissions is the sum of complex COPD-related, minor COPD-related, pneumonia, and COPD-related hospital/rehabilitation in the home admissions. AUD = Australian dollars, COPD=chronic obstructive pulmonary disease.

## Data Availability

The original data presented in this study are included in the article and Supplementary Material.

## Supplementary Materials

The following supporting information can be downloaded at https://www.mdpi.com/article/10.3390/jcm14113894/s1, Table S1: Summary of breathlessness intervention service (BLIS) program intervention as planned; Table S2: Person living with breathlessness and carer-reported outcome measures; Table S3: Completion of pre/post-BLIS program self-report outcome measures; Table S4: Multidimensional Dyspnea Profile scores for all items in people living with breathlessness at pre-program compared with post-program and follow-up; Table S5: Breathlessness threat (B-IPQ), DASS-21, and AQOL-8D: pre-program compared with post-program and follow-up; Supplement S1: Example Breathlessness Management Plan blank template; Supplement S2: Unmet support needs to be identified and addressed by the BLIS program in six carers.

## Abbreviations

The following abbreviations are used in this manuscript:

AQoL8-D	Assessment of Quality of Life
B-IPQ	Brief Illness Perception Questionnaire (breathlessness version)
BLIS	Breathlessness Intervention Service
BTF	Breathing Thinking Functioning
CBIS	Cambridge Breathlessness Intervention Service
CI	Confidence Interval
COPD	Chronic Obstructive Pulmonary Disease
CRQ	Chronic Respiratory Questionnaire
DASS-21	Depression, Anxiety, and Stress Scale
DRG	Diagnosis-Related Group
FEV_1_%pred	Forced expiratory volume in one second percentage predicted
HREC	Human Research Ethics Committee
MDP	Multidimensional Dyspnea Profile
NHS	National Health Service
QALY	Quality-Adjusted Life Years
RNP	Respiratory Nurse Practitioner
UK	United Kingdom
ZBI-12	Zarit Burden Interview

## References

[1] van Dijk M., Gan C.T., Koster T.D., Wijkstra P.J., Slebos D.J., Kerstjens H.A.M., van der Vaart H., Duiverman M.L. (2020). Treatment of severe stable COPD: The multidimensional approach of treatable traits. ERJ Open Res..

[2] Miravitlles M., Ribera A. (2017). Understanding the impact of symptoms on the burden of COPD. Respir. Res..

[3] Johnson M.J., Yorke J., Hansen-Flaschen J., Lansing R., Ekström M., Similowski T., Currow D. (2017). Towards an expert consensus to delineate a clinical syndrome of chronic breathlessness. Eur. Respir. J..

[4] Disler R., Green A., Luckett T., Newton P., Inglis S., Currow D., Davidson P. (2014). Experience of advanced chronic obstructive pulmonary disease: Metasynthesis of qualitative research. J. Pain Symptom Manag..

[5] Reitzel T., Bergmann A., Schloesser K., Pauli B., Eisenmann Y., Randerath W., Tuchscherer A., Frank K., Simon S.T., Pralong A. (2022). The experience of episodic breathlessness from the perspective of informal caregivers: A qualitative interview study. Ann. Palliat. Med..

[6] Dzingina M., Reilly C., Bausewein C., Jolley C., Moxham J., McCrone P., Higginson I., Yi D. (2017). Variations in the cost of formal and informal health care for patients with advanced chronic disease and refractory breathlessness: A cross sectional secondary analysis. Palliat. Med..

[7] Brighton L.J., Miller S., Farquhar M., Booth S., Yi D., Gao W., Bajwah S., Man W.D., Higginson I.J., Maddocks M. (2019). Holistic services for people with advanced disease and chronic breathlessness: A systematic review and meta-analysis. Thorax.

[8] Spathis A., Reilly C.C., Bausewein C., Reinke L.F., Romero L., Smallwood N.E., Ekström M., Holland A.E. (2024). Multicomponent services for symptoms in serious respiratory illness: A systematic review and meta-analysis. Eur. Respir. Rev..

[9] Holland A.E., Spathis A., Marsaa K., Bausewein C., Ahmadi Z., Burge A.T., Pascoe A., Gadowski A.M., Collis P., Jelen T. (2024). European Respiratory Society Clinical Practice Guideline on symptom management for adults with serious respiratory illness. Eur. Respir. J..

[10] Seidl H., Schunk M., Le L., Syunyaeva Z., Streitwieser S., Berger U., Mansmann U., Szentes B.L., Bausewein C., Schwarzkopf L. (2023). Cost-effectiveness of a specialized breathlessness service versus usual care for patients with advanced diseases. Value Health.

[11] Farquhar M.C., Prevost A.T., McCrone P., Brafman-Price B., Bentley A., Higginson I.J., Todd C., Booth S. (2014). Is a specialist breathlessness service more effective and cost-effective for patients with advanced cancer and their carers than standard care? Findings of a mixed-method randomised controlled trial. BMC Med..

[12] Schunk M., Le L., Syunyaeva Z., Haberland B., Tänzler S., Mansmann U., Schwarzkopf L., Seidl H., Streitweiser S., Hofmann M. (2021). Effectiveness of a specialised breathlessness service for patients with advanced disease in Germany: A pragmatic fast-track randomised controlled trial (BreathEase). Eur. Respir. J..

[13] Mooren K., Wester D., Kerstjens H., Bergkamp E., Spathis A., Engels Y. (2022). Filling the gap: A feasibility study of a COPD-specific breathlessness service. COPD.

[14] Drury A., Goss J., Afolabi J., McHugh G., O’Leary N., Brady A. (2023). A mixed methods evaluation of a pilot multidisciplinary breathlessness support service. Eval. Rev..

[15] Qian M.Y., Politis J., Thompson M., Wong D., Le B., Irving L., Smallwood N. (2018). Individualized breathlessness interventions may improve outcomes in patients with advanced COPD. Respirology.

[16] Luckett T., Roberts M.M., Smith T., Swami V., Cho J.G., Wheatley J.R. (2020). Patient perspectives on how to optimise benefits from a breathlessness service for people with COPD. NPJ Prim. Care Respir. Med..

[17] Lancaster G.A., Thabane L. (2019). Guidelines for reporting non-randomised pilot and feasibility studies. Pilot Feasibility Stud..

[18] Yang I.A., George J., McDonald C.F., McDonald V., Ordman R., Goodwin A., Smith B., McNamara R., Zwar N., Dabscheck E. The COPD-X Plan: Australian and New Zealand Guidelines for the Management of Chronic Obstructive Pulmonary Disease 2024. Version 2.76. https://copdx.org.au/copd-x-plan/.

[19] Booth S., Burkin J., Moffat C., Spathis A. (2014). Managing Breathlessness in Clinical Practice.

[20] Spathis A., Booth S., Moffat C., Hurst R., Ryan R., Chin C., Burkin J. (2017). The Breathing, Thinking, Functioning clinical model: A proposal to facilitate evidence-based breathlessness management in chronic respiratory disease. NPJ Prim. Care Respir. Med..

[21] Battersby M.W., Ask A., Reece M.M., Markwick M.J., Collins J. (2001). A case study using the “Problems and Goals Approach” in a coordinated care trial: SA HealthPlus. Aust. J. Public Health.

[22] Ewing G., Grande G. (2013). Development of a Carer Support Needs Assessment Tool (CSNAT) for end-of-life care practice at home: A qualitative study. Palliat. Med..

[23] Saunderson E. (2014). Family and friends test. Br. J. Gen. Pract..

[24] Banzett R.B., O’Donnell C.R., Guilfoyle T.E., Parshall M.B., Schwartzstein R.M., Meek P.M., Gracely R.H., Lansing R.W. (2015). Multidimensional Dyspnea Profile: An instrument for clinical and laboratory research. Eur. Respir. J..

[25] Williams J.E., Singh S.J., Sewell L., Guyatt G.H., Morgan M.D. (2001). Development of a self-reported Chronic Respiratory Questionnaire (CRQ-SR). Thorax.

[26] Broadbent E., Petrie K.J., Main J., Weinman J. (2006). The brief illness perception questionnaire. J. Psychosom. Res..

[27] Henry J.D., Crawford J.R. (2005). The short-form version of the Depression Anxiety Stress Scales (DASS-21): Construct validity and normative data in a large non-clinical sample. Br. J. Clin. Psychol..

[28] Richardson J.R., Iezzi A., Khan M.A., Maxwell A. (2014). Validity and reliability of the Assessment of Quality of Life (AQoL)-8D multi-attribute utility instrument. Patient.

[29] Gratão A.C., Brigola A.G., Ottaviani A.C., Luchesi B.M., Souza É.N., Rossetti E.S., de Oliveira N.A., Terassi M., Pavarini S.C. (2019). Brief version of Zarit Burden Interview (ZBI) for burden assessment in older caregivers. Dement. Neuropsychol..

[30] Australian Institute of Health and Welfare [AIHW] 2023 Hospitals Glossary. https://www.aihw.gov.au/reports-data/myhospitals/content/glossary.

[31] (2019). Independent Health and Aged Care Pricing Authority [IHACPA] (Formerly Independent Hospital Pricing Authority) (2019) Australian Refined Diagnosis Related Groups Version 10.0 Final Report. https://www.ihacpa.gov.au/sites/default/files/2022-08/AR-DRG%20Version%2010.0%20Final%20Report_0.pdf.

[32] Ronk F.R., Korman J.R., Hooke G.R., Page A.C. (2013). Assessing clinical significance of treatment outcomes using the DASS-21. Psychol. Assess..

[33] Booth S., Farquhar M., Gysels M., Bausewein C., Higginson I.J. (2006). The impact of a breathlessness intervention service (BIS) on the lives of patients with intractable dyspnea: A qualitative phase 1 study. Palliat. Support Care.

[34] Hill K., Hug S., Smith A., O’Sullivan P. (2024). The role of illness perceptions in dyspnoea-related fear in chronic obstructive pulmonary disease. J. Clin. Med..

[35] Williams M.T., Lewthwaite H., Paquet C., Cafarella P., Frith P. (2023). Pulmonary rehabilitation with and without a cognitive behavioral intervention for breathlessness in people living with chronic obstructive pulmonary disease: Randomized controlled trial. J. Clin. Med..

[36] Higginson I.J., Gao W., Jackson D., Murray J., Harding R. (2010). Short-form Zarit Caregiver Burden Interviews were valid in advanced conditions. J. Clin. Epidemiol..

[37] Micklewright K., Farquhar M. (2022). Face and content validity of the Carer Support Needs Assessment Tool (CSNAT), and feasibility of the CSNAT intervention, for carers of patients with chronic obstructive pulmonary disease. Chronic Illn..

[38] Ekström M., Bornefalk H., Sköld C.M., Janson C., Blomberg A., Sandberg J., Bornefalk-Hermansson A., Currow D., Johnson M., Sundh J. (2021). Minimal clinically important differences for Dyspnea-12 and MDP scores are similar at 2 weeks and 6 months: Follow-up of a longitudinal clinical study. Eur. Respir. J..

[39] Alqahtani J.S., Oyelade T., Aldhahir A.M., Mendes R.G., Alghamdi S.M., Miravitlles M., Mandal S., Hurst J. (2021). Reduction in hospitalised COPD exacerbations during COVID-19: A systematic review and meta-analysis. PLoS ONE.

[40] So J.Y., O’Hara N.N., Kenaa B., Williams J.G., deBorja C.L., Slejko J.F., Zafari Z., Sokolow M., Zimand P., Deming M. (2021). Population decline in COPD admissions during the COVID-19 pandemic associated with lower burden of community respiratory viral infections. Am. J. Med..

