# Supplementary material for: Translating a Home-Based Breathlessness Service: A Pilot Study of Feasibility, Person-Reported, and Hospital Use Outcomes
Source: J Clin Med. 2025 Jun 1;14(11):3894. doi: 10.3390/jcm14113894 (PMC12156291; doi:10.3390/jcm14113894)
Supplement: Supplementary file 1 [file jcm-14-03894-s001.zip › jcm-3654127-supplementary.pdf]

Supplementary Materials

Table S1: Summary of Breathlessness Intervention Service (BLIS) program intervention as planned (Respiratory Nurse Practitioner [RNP] and /or physiotherapist undertake the home visits and follow up phone calls).

Week /Visit	Details
Week 1<br>Visit 1	**Aims:**<br>Establish the problem from the patient and carers' standpoint, the key priorities the patient and carer wish to work on, and expectations about the program.<br>Appreciate which of the "breathing/thinking/functioning" vicious cycles predominate in perpetuating or worsening patient's breathlessness.<br>Quantify the experience of the symptom and its impact so that response to management can be determined.<br>Give something they can change from the first visit: action plan for breathlessness including fan/position/focus on breath out<br><br>ASSESSMENT GUIDE VISIT 1<br>1. Look for reversible causes: speed, onset, temporal pattern of breathlessness (eg sudden onset)<br>Ask about associated symptoms (pain, sputum, wheeze as relevant)<br>2. Breathing:<br>• find out behaviour of the breathlessness; precipitating and relieving factors, (eg influenced by body position, type of exertion, room temperature, medications)<br>• Words and phrases used to describe the sensations<br>• Use NRS scale for unpleasantness: how breathless are you (right now/last 2 weeks?) on a scale of 0-10 where 0 is not breathless and 10 is the worst breathlessness you can imagine?<br>3. Thinking<br>• Ask about patient's understanding of both the causes and consequences of their symptoms and underlying disease (eg some patients believe that breathlessness itself is causing damage to their body)<br>• Find out about their emotional reaction, in terms of anxiety, mood, panic<br>• Use NRS scale to determine magnitude of predominant emotional response. Eg if anxiety, How anxious are you (right now/last 2 weeks?) on a scale of 0-10 where 0 is not anxious at all and 10 is the worst anxiety you can imagine?<br>4. Functioning<br>• Establish previous and current levels of activity/exercise tolerance inside and outside the home<br>• Try to understand social limitations imposed by the symptom, in terms of altered roles, loss of friendships, inability to do shopping etc<br>• Find out if equipment is being used or needed for gait or ADLs<br>• Consider the role of the carer in terms of additional responsibilities/burden<br>5. Expectations and priorities<br>• Find out expectations about how their problems may change over time. Try to ascertain their expectations about how a health professional may help<br>• Find out the patient's priorities/goals: for example being able to walk the dog again; being able to sleep well at night, to know they can abort a panic attack if it arose<br>6. Existing coping strategies<br>• Ask specifically what they do when they are feeling particularly breathless to try to help the symptom settle quickly. This may include medication.<br>• All patients will have discovered ways of trying to manage their symptoms, even if apparently not coping very well.<br>• How confident do they feel in being able to manage their breathlessness when it happens? (NRS scale)<br><br>MANAGEMENT GUIDE VISIT 1

	Decide which 1 or 2 of the “breathing, thinking, functioning” vicious cycles is the greatest problem and use this to focus initial management<br>This is most likely to involve quick acting effective strategies such as<br>Explaining and using the fan<br>Developing a short action plan for breathlessness (fan, positioning, recovery breathing technique)<br>Teach an anxiety reduction technique: eg mantra as part of action plan/relaxation/visualisation technique<br>Can include challenging unhelpful thoughts/beliefs related to the BTF vicious cycles<br><br>Having identified the patient’s own ways of managing breathlessness, explicitly praise the adaptive strategies; enhance sense of resilience and problem solving ability.<br>Manage patient expectations, explaining that improvement take small, incremental steps over many weeks, requires engagement and practice.<br><br>Formulate a plan using SMART goals<br><br>CARER: As they wish may be present with the participant at the visit. Alternatively it may be useful to speak by phone or meet with the carer individually. Facilitating support of the carer is vitally important and this may mean facilitating access to other services as well as the BLIS program.
Week 2<br>Visit 2	Follow up visits will re-assess, review and if required modify initial management strategies and plan.<br>Add further strategies that address other aspects of breathing/thinking/functioning as indicated.<br>The second visit may be the place for goals/priorities/interventions around exercise and activity promotion, eg using pedometer to incrementally increase exercise; NMES or strength training as a bridge to endurance exercise; activity pacing<br><br>CARER: Needs assessment using CSNAT
Week 4<br>Visit 3<br><u>if indicated</u>	Follow up visits and contacts will re-assess, review and if required modify initial management strategies.<br>Add further strategies that address other aspects of breathing/thinking/functioning as indicated.<br>Liaise with other services that are suitable for long term support or to further meet the patient’s needs: such as referral to pulmonary rehabilitation; palliative care services; community-based exercise or social program etc. and refer<br>Review achievement of SMART goals<br><br>The BLIS program is generally short term (6-8 weeks) promoting self-management and integration into community supports. However there may be participants who would benefit from planned review and/or re-referral to the program.
Week 5<br>Follow up 1<br>(phone)	
Week 7<br>Follow up 2<br>(phone)	
Week 8<br>Follow up 3<br>(phone) if indicated.	Post out booklets (participants and carers) with these questionnaires to complete BLIS study questionnaire booklet(s), patient and carer.

Supplement S1: Example Breathlessness Management Plan blank template

BREATHLESSNESS MANAGEMENT PLAN

Breathlessness Intervention Service (BLIS)

<Date>
<Name>

<Diagnosis>

<Specialist>

When feeling breathless remember the following:

1. <step-by-step instructions for the most helpful non-drug breathlessness strategy for that person>
2. <step-by-step instructions for additional helpful non-drug breathlessness strategy for that person>
3. If steps 1 and 2 are not helping you recover, use your medication for breathlessness <individualised instructions for reliever medication etc as prescribed>

If breathlessness continues:

1. Continue to rest in a supported position, focussing on the breath out

2. If no relief after 30 minutes use your medication for breathlessness again:

< individualised instructions for reliever medication etc as prescribed>

If breathlessness continues, more than expected and you think it is because of a flare up of your condition:

1. Read your COPD Action Plan and

   < individualised points based on COPD Action Plan, eg regarding increased/ additional medication use, airway clearance if prescribed> and

   - Make an appointment to see your GP

If no relief and breathlessness persists consider calling for more help:

1. Call 000 for an ambulance

REMEMBER YOU CAN RECOVER FROM BREATHLESSNESS, BUT YOU CAN ALSO CALL FOR HELP AT ANY STAGE IF YOU NEED TO

You can also consider the following;

< list one to four additional highly relevant, specific, individualised non-drug strategies that were effective in short, medium and/or longer term to manage breathlessness for that person>

Table S2: Person living with breathlessness and carer reported outcome measures

Questionnaires were printed and combined into separate booklets for the person living with breathlessness or carer, respectively. Participants were offered options for both self-completion and phone-based interviewer-assisted completion.

**Instrument**	**Number of items**	**Focal period**
**Person living with breathlessness reported outcomes**		
Multidimensional Profile for Dyspnea (MDP) [24]	11	past 2 weeks
Chronic Respiratory Questionnaire- Self Administered, Mastery subscale [25]	4	past 2 weeks
Visual analogue scale (VAS) for perceived ability to manage/ live with breathlessness over the past week (0 = not able to manage at all, 10 = able to manage extremely well)	1	past week
Brief Illness Perception Questionnaire -Breathlessness version [26]	8	not specified
Depression Anxiety Stress Scales (DASS-21) [27]	21	past week
Assessment of Quality of Life (AQoL-8D) [28]	35	past week
**Carer reported outcomes**		
Zarit Burden Interview (ZBI-12) [29]	12	past 2 weeks
Visual analogue scale (VAS) for perception of the ability of the person they were caring for to manage/ live with their breathlessness (0 = not able to manage at all, 10 = able to manage extremely well)	1	past week
Visual analogue scale (VAS) for perception of own ability to manage and live with the person they were caring for with breathlessness (0 = not able to manage at all, 10 = able to manage extremely well)	1	past week
Depression Anxiety Stress Scales (DASS-21) [27]	21	past week

Table S3: Completion of pre/post BLIS program self-report outcome measures (n=15 people with COPD)

Questionnaire item	Completion pre-program (n=15)	Completion post-program (n=15)*	Comment
Multidimensional Dyspnoea Profile			
A1 scale	15 (100%)	15 (100%)	At times direction of rating was at odds with qualitative interview feedback
Sensory Qualities forced choice	14 (93%)	7 (47%)	Missing responses left blank when participant could not make a forced choice/none applied/none best described
Sensory Qualities intensity	15 (100%)	13 (87%)	Missing responses were blank. At times direction of rating was at odds with qualitative interview feedback
Emotional Response intensity	15 (100%)	11(73%)	One or more responses were blank
CRQ-Self Administered Survey (Mastery subscale)	15 (100%)	15 (100%)	One participant including different responses for "good days" and "bad days" highest values used
Ability to manage breathlessness over last week (VAS 0-10)	14 (93%)	15 (100%)	One blank at pre-program
Brief Illness Perception Questionnaire (BIPQ, breathlessness version)			
Numerical rating scale items (n=7)	15 (100%)	13 (87%)	Two participants left one item blank at post-program, stating "did not understand this question"
BIPQ cause item (free text)<br>1 cause<br>2 unique causes<br>3 unique causes	 <br>14 (93%)<br>8 (53%)<br>6 (40%)	 <br>14 (93%)<br>10 (67%)<br>8 (53%)	One completely blank (different participants) at each of pre and post
Depression Anxiety Stress Scales (DASS21)	15 (100%)	15(100%)	
Assessment of Quality of Life (AQoL-8D)	14(93%)	15(100%)	One pre-program response missed all items in "relationships" subscale, could not be imputed (thus no summed score).

*3 post-program responses were posted back only, with no telephone debrief at participants' request
CRQ=Chronic Respiratory Questionnaire

Table S4: Multidimensional Dyspnea Profile scores for all items in people living with breathlessness at pre-program, compared with post-program and follow-up (three and six months post program). Values mean (standard deviation, SD) except where indicated as median (interquartile rage, IQR). **Bold text values** indicate 95% confidence interval of difference did not cross zero.

	pre-program week 0<br>n=15	post-program week 9<br>n=15	Difference post from pre mean(SD) 95% CI n=15	3mths post-program<br>n=11	Difference 3mth from pre mean(SD) 95% CI n=11	6mths post-program<br>n=10	Difference 6mth from pre mean(SD) 95% CI n=10
**MDP A1 scale (unpleasantness/discomfort)**<br>0=neutral, 10=unbearable	4.9(2.3)	3.9(1.8)	**-1.0 (1.8)**<br>**-2.0, -0.1**	4.7(2.4)	-1.2 (1.9)<br>(-2.5, 0.1)	5.0(2.1)	**-1.1(1.4)**<br>**(-2.1, -0.1)**
**Sensory Qualities intensity**<br>0=none, 10=as intense as I can imagine	median(IQR)	median(IQR)<br>n=13		median (IQR)		median (IQR)	
My breathing requires muscle work ***or*** effort	5.0(6.0)	3.0(5.0)	-1.1(2.9)<br>-2.8, 0.7<br>n=13	1.5(5.0)<br>n=10	-1.4(2.1)<br>-2.8, 0.7<br>n=10	3.5(7.0)<br>n=10	-0.9 (2.4)<br>-2.6, 0.8<br>n=10
I am not getting enough air ***or*** I am smothering ***or*** I feel hunger for air	2.0(7.0)	1.0(5.0)	0.1(3.4)<br>-2.0, 2.1<br>n=13	3.0(6.0)<br>n=10	0.8(2.3)<br>-0.8, 2.5<br>n=10	1.0(6.0)<br>n=8	-0.8(3.5)<br>-3.6, 2.1<br>n=8
My chest and lungs feel tight ***or*** constricted	3.0(6.0)	1.0(4.0)	-1.0(3.2)<br>-3.0, 1.0<br>n=13	5.0(6.0)<br>n=11	-0.5(2.0)<br>-1.8, 0.8<br>n=11	3.0(3.0)<br>n=9	-0.5(2.4)<br>-2.3, 1.3<br>n=9
My breathing requires mental effort ***or*** concentration	3.0(7.0)	1.5(4.0)<br>n=12	-0.8(2.9)<br>-2.6, 1.1<br>n=12	1.5(6.0)<br>n=10	-0.5(2.2)<br>-2.1, 1.1<br>n=10	2.5(6.0)<br>n=10	-0.6 (3.0)<br>-2.7, 1.6<br>n=10
I am breathing a lot	5.0(6.0)	3.0(6.0)	-1.5(3.4)<br>-3.6, 0.5<br>n=13	4.0(5.0)<br>n=10	-0.7(4.5)<br>-4.1, 2.7<br>n=10	1.5(7.0)<br>n=8	-2.8(4.8)<br>-6.9, 1.1<br>n=8
SQ intensity of individual's forced choice category	6.0(3.0)<br>n=14	4.5(5.0)<br>n=8	-0.5(2.5)<br>-2.6, 1.6<br>n=8	6.5(3.0)<br>n=8	-0.1(2.4)<br>-2.1, 1.8<br>n=8	3.5(4.0)<br>n=8	-1.9(4.0)<br>-5.2, 1.5<br>n=8
**Emotional Response**<br>0=none, 10=most I can imagine	median(IQR)<br>n=15	median(IQR)<br>n=14		median (IQR)<br>n=11		median(IQR)<br>n=10	
My breathing sensations make me feel depressed	0(8.0)	0(6.0)<br>n=13	-1.3(2.8)<br>-3.1, 0.3<br>n=13	2.0(2.7)	0.5(3.9)<br>-2.1, 3.2<br>n=11	0(2.0) n=9	-1.7(3.1)<br>-4.0, 0.7<br>n=9
My breathing sensations make me feel anxious	3.0(6.0)	1.0(3.0)<br>n=13	**-1.5(3.6)**<br>**-1.5, -3.7**<br>n=13	1.0(2.8)	-0.9(3.6)<br>-3.3, 1.5<br>n=11	1.0(6.0)<br>n=9	-1.8(3.2)<br>-4.3, 0.7<br>n=9
My breathing sensations make me feel frustrated	4.0(8.0)	2.5(4.0)<br>n=12	-1.4(3.2)<br>-3.5, 0.6<br>n=12	5.0(5.0)	0.4(3.7)<br>-2.0, 3.0<br>n=11	3.0(7.0)	-0.6(2.7)<br>-2.5, 1.3<br>n=10
My breathing sensations make me feel angry	0(8.0)	0(1.0)	-2.0(3.5)<br>-4.1, 0.1<br>n=14	1.0(7.0)<br>n=10	0.1 (2.2)<br>-1.5, 1.7<br>n=10	0(6.0)	-0.9(1.3)<br>-1.8, 0.0<br>n=10
My breathing sensations make me feel afraid	0(5.0)	0(1.0)<br>n=13	-0.3(3.1)<br>-2.2, 1.5<br>n=13	1.0(5.0)	0.9(1.9)<br>-0.4, 2.2<br>n=11	0(3.0)<br>n=9	0.2(1.7)<br>-1.1, 1.5<br>n=9

Table S5: Breathlessness threat (B-IPQ), DASS-21 and AQOL-8D: pre-program, compared with post-program and follow-up (three and six month) scores. Values mean (standard deviation, SD) except where indicated as median (interquartile rage, IQR). **Bold text** values indicate 95% confidence interval of difference did not cross zero.

	pre-program week 0<br>n=15	post-program week 9<br>n=15	Difference post from pre, mean (SD) 95% CI<br>n=15	3mths post-program<br>n=11	Difference 3mth from pre mean(SD) 95% CI<br>n=11	6mths post-program<br>n=10	Difference 6mth from pre mean(SD) 95% CI<br>n=10
**Brief Illness Perception Questionnaire,** higher score reflects a more threatening viewpoint							
**Total score**	37.3(7.9)	32.3(10.7)	**-5.1(8.3)**<br>**-9.7, -0.5**	32.1(8.3)	**-7.2 (8.5)**<br>**-12.9, -1.5**	32.5(11.5)	-6.4(12.0)<br>-15.0, 2.2
**Consequences**: How much does breathlessness affect your life?<br>0=no affect, 10 severely affects	7.4(1.2)	5.7(3.1)	**-1.7(2.9)**<br>**-3.3, -0.1**	4.9 (2.9)	-2.5(2.3)<br>-4.0, -0.9	6.3(2.7)	-0.9(2.6)<br>-2.7, 1.0
**Timeline**: How long do you think your breathlessness will continue?<br>0=a very short time, 10=forever	10.0(1.0)<br>median(IQR)	10.0(1.0)<br>median(IQR)	0.2 (1.6) -<br>0.6, 1.1	10.0(2.0)<br>median(IQR)	-0.4(0.7)<br>-0.8, 0.9	10.0(1.0)<br>median(IQR)	-0.3(2.0)<br>-1,7, 1.1
**Personal control**<br>0=absolutely no control 10=extreme amount of control	6.5(2.5)	7.2(2.1)	0.7(2.7)<br>-0.7, 2.2	7.1(1.4)	0.9(2.9)<br>-1.0, 2.8	6.8(1.8)	1.1(2.7)<br>-0.8, 3.0
**Treatment control**: How much do you think your treatment can help your breathlessness? 0=not at all, 10=very helpful	8.0(4.0)<br>median(IQR)	8.5(3.0)<br>median(IQR)	0.4(2.4)<br>-1.1, 1.8<br>n=14	8.0(2.0)<br>median(IQR)<br>n=10	1.1(2.4)<br>-0.6, 2.8<br>n=10	8.0(3.0)<br>median(IQR)	-0.1(2.5)<br>-1.9, 1.6
**Concern**<br>0=not at all concerned, 10=extremely concerned	7.3(2.5)	5.6(3.1)<br>n=14	**-2.0(2.1)**<br>**-3.3, -0.7**<br>n=14	6.3(2.5)	-1.9(3.0)<br>-3.9, 0.1	5.6(3.0)	-1.9(4.3)<br>-5.0, 1.2
**Coherence**: Understand your breathlessness 0=Not at all, 10=very clearly	10.0(3.0)<br>median(IQR)	9.0(3.0)<br>median(IQR)	-0.3(1.9)<br>-1.4, 0.7	9.0(2.0)<br>median(IQR)	-0.1(2.1)<br>-1.5, 1.3	9.0(2.0)<br>median(IQR)	0.1(1.0)<br>-0.6, 0.8
**Emotional affect**<br>0=not at all affected emotionally<br>10=extremely affected emotionally	6.4(3.2)	4.7(3.0)	**-1.7(2.4)**<br>**-3.1, -0.4**	4.8(3.0)	-1.5(2.6)<br>-3.3, 0.2	5.5(2.9)	**-2.2(2.4)**<br>**-3.9, -0.5**
	pre-program week 0<br>n=15	post-program week 9<br>n=15	Difference post from pre, mean (SD) 95% CI,<br>n=15	3mths post-program<br>n=11	Difference 3mth from pre mean(SD) 95% CI,<br>n=11	6mths post-program<br>n=10	Difference 6mth from pre mean(SD) 95% CI,<br>n=10
**DASS-21** (pre, post and follow up scores all reported as median (IQR), lower score is better							
**Depression subscale**	6(8)	6(12)	-1.5(5.5)<br>-4.5, 1.6	6(8)	1.2 (7.7)<br>-3.9, 6.5	4(32)	1.8(8.9)<br>-4.6, 8.2
**Anxiety subscale**	12(18)	10(14)	**-4.1(6.0)**<br>**-7.5, -0.8**	10(16)	-1.8(5.8)<br>-5.6, 2.1	9(20)	-2.4(7.2)<br>-7.6, 2.7
**Stress subscale**	10(12)	6(20)	0.5 (7.5)<br>-3.6, 4.7	6(20)	2.1(9.3)<br>-4.1, 8.4	13(28)	3.5(7.7)<br>-2.0, 9.1

	pre-program week 0<br><br>n=15	post-program week 9<br><br>n=15	Difference post from pre, mean (SD) 95% CI,<br>n=15	3mths post-program<br><br>n=11	Difference 3mth from pre mean(SD) 95% CI,<br>n=11	6mths post-program<br><br>n=10	Difference 6mth from pre mean(SD) 95% CI,<br>n=10
**AQoL 8D** psychometric (raw) score, lower score is better unless indicated							
**Total** (additive score)	80.2(19.5)	77.3(19.9)<br>n=14	-2.9(6.7)<br>-6.8, 0.9<br>n=14	80.8(23.4)<br>n=10	-1.4 (10.9)<br>-9.2, 6.4<br>n=10	86.2(24.1)<br>n=9	2.3*(11.8)<br>-6.2, 11.9<br>n=9
**Total** (standardized score, higher is better)	68.3(13.9)	70.7(14.5)<br>n=14	2.4(4.9)<br>-0.4, 5.3<br>n=14	68.5(16.0)	1.1(7.3)<br>-3.7, 6.0	64.8(16.5)	-2.1# (7.9)<br>-7.7, 3.6
**Independent living dimension score**	12.7(2.8)	11.7(2.9)	-1.0(1.9)<br>-2.1, 0.1	13.0(3.5)	-0.4(3.3)<br>-2.6, 1.9	12.5(3.1)	-1.0(3.3)<br>-3.4, 1.4
**Senses dimension score**	6.2(1.8)	5.9(1.5)	-0.2(1.6)<br>-1.1, 0.6	6.2(2.2)	0.3(2.3)<br>-1.3, 1.8	5.8(1.5)	-0.3 (1.6)<br>-1.4, 0.8
**Pain dimension score**	7.0(3.7)	6.5(3.4)	-0.5(1.5)<br>-1.3, 0.3	6.5(3.8)	-0.7(2.6)<br>-2.5, 1.1	5.6(3.1)	-1.0(1.9)<br>-2.3, 0.3
**Mental health dimension score**	17.2(5.6)	18.1(6.5)	0.9 (2.7)<br>-0.6, 2.3	18.0(6.9)	1.3(3.2)<br>-0.9, 3.4	19.1(6.9)	0.9(2.2)<br>-0.7, 2.5
**Happiness dimension score**	9.3(2.6)	8.9(2.6)	-0.4(1.8)<br>-1.4, 0.6	9.4(3.1)	-0.4 (1.3)<br>-1.2, 0.5	10.5(3.6)	1.0(3.0)<br>-1.1, 3.1
**Self worth dimension score**	6.3(2.5)	6.3(2.4)	-0.1(1.4)<br>-0.8, 0.7	6.2(2.7)	-0.5(1.2)<br>-1.2, 0.4	7.0(3.0)	0.5(2.5)<br>-1.3, 2.3
**Coping dimension score**	7.7(1.8)	6.6(1.8)	**-1.1(1.6)**<br>**-2.0, -0.3**	6.9(2.2)	**-1.2(1.7)**<br>**-2.3, -0.7**	7.7(3.1)	-0.1(2.6)<br>-1.9, 1.8
**Relationships dimension score**	14.6(4.6)	14.3(4.8)<br>n=14	-0.4(2.4)<br>-1.7, 1.0<br>n=14	15.2(5.2)<br>n=10	0.1(3.1)<br>-2.1, 2.3<br>n=10	17.8(5.9)<br>n=9	2.2(3.7)<br>-0.6, 5.0<br>n=9

Supplement S2: Unmet support needs identified and addressed by BLIS program in 6 carers

During the BLIS Program, carers had the opportunity to discuss and identify any unmet support needs that could be addressed. Two of 6 carers were still working (part-time and full-time capacity). Both these carers discussed potential support needs in the presence of the participant, indicated that they were managing the home care, scored a "no", or "a little more" on the Carer Support Needs Assessment Tool [22], indicating a need at the time, but not warranting assistance, and declined further intervention, supports, or further discussions with the RNP. The remaining four carers chose to complete and discuss the carers assessment without the participant present and engaged in the ongoing process of need identification and taking steps to address the reported need(s).

Unmet support needs identified and addressed by BLIS program in 6 carers

**Need**	**Frequency**	**Action taken by the BLIS team**
Dealing with emotions and fears	2	Services info provided for carer to engage when ready
Knowing what to expect in the future	2	RNP discussions with carers about disease trajectory
Understanding illness	2	RNP discussions with carers about pathophysiology and management of disease<br>Community Respiratory nursing case management contacted to address request for regular home nursing input and for contact point for any concerns about participant
Respite and / or emergency care needs	1	Coordinated Care Information Pack provided<br>Coordinated via aged care package - in home respite<br>Registered Commonwealth Respite Centre for future crisis access
Home based physiotherapy	1	Coordinated via aged care package services
Personal care supports	1	Coordinated via aged care package services
Poor communication from medical officer about treatment	1	RNP attended outpatient review for support<br>Carer developed list of questions to ask
Looking after own health	1	National aged care referral for carers personal care needs assessment and formal supports at home

RNP=Respiratory Nurse Practitioner
